# Self-reflection on competence: metacognitive process and barriers of self-assessment in psychologists

**DOI:** 10.1007/s10459-025-10434-7

**Published:** 2025-05-01

**Authors:** Stephanie E. Banner, Adam J. Rock, Suzanne M. Cosh, Nicola Schutte, Kylie Rice

**Affiliations:** School of Psychology, University of New England, Armidale, Australia

**Keywords:** Self-assessment, Self-reflection, Competency, Metacognition, Continuing education

## Abstract

Psychologists, like many health professionals, must effectively self-assess their competencies across the career trajectory in order to practice ethically and within the bounds of their knowledge and skills. Given this, research is needed to explore the cognitive process of self-assessment, and the utility of a structured measure for psychologists. This study aimed to explore: 1) how psychologists self-assess their competencies, 2) what value a structured measure has for self-assessment, and 3) the barriers or limitations to using a structured measure for self-assessment. Twelve registered psychologists were interviewed, and reflexive thematic analysis was applied. Themes were identified in the following two broad areas: 1) metacognitive processes and helpful cognitive strategies that aid self-assessment and the use of a structured self-assessment measure, and 2) barriers to self-assessment including unhelpful metacognitions about both the process of self-assessment itself, and the self. One theme and three sub-themes were identified to explain the metacognitive processes and helpful cognitive strategies described by participants, and three themes were identified pertaining to barriers to self-assessment. The current research extends the existing literature through incorporating the voice of psychologists in understanding the metacognitive processes and barriers of self-assessment, including structured self-assessment. This research has implications for health science practitioners, supervisors, educators and research.

Across recent decades, there has been a marked shift in the allied health and medical fields towards a competency-based approach to assessment, whereby governing bodies emphasise the outputs of training in terms of competency development, rather than input of hours by students (Gonsalvez et al., 2016). Stevens et al. (2017) define competence as “an individual’s professional skills across numerous domains” (p. 175), with competencies being “the particular skill that sit within these domains” (p. 175). This “competency revolution” (Nicholson Perry et al., 2017) has led to the delineation of competencies for various health disciplines across the world, such as midwifery (e.g., the International Confederation of Midwives, 2024), pharmacy (e.g. Pharmacy Council of New Zealand, 2015) and dentistry (e.g. American Dental Education Association, 2008) amongst others. The movement to competency-based training and assessment has included the discipline of psychology, as regulatory bodies across the world such as the Australian Psychology Accreditation Council (2019) and the British Psychological Society (2019) have delineated the competencies required of those psychologists registered within their jurisdiction. As a result, training programs aim to ensure all trainee psychologists are deemed competent across all required psychological competencies.

However, the competency approach to practice needs to extend beyond initial training. Models of psychological competence, such as Rodolfa et al.’s (2005) cube model, identify that competencies continue to develop beyond formal training and further into the career, which they describe as “continuing competency”. Professional development requirements of registered psychologists in many countries implicate psychologists to maintain currency of knowledge and skills, extending the need for practitioners to adopt a competency-based approach beyond training. For example, the Australian Psychological Society’s Code of Ethics (2007) identifies competence as an ethical obligation, whereby psychologists must “ … maintain appropriate skills and learning to their areas of professional practice” and “continuously monitor their professional functioning” (p. 19).

Maintaining competency across the career is considered important in the context of ensuring psychologists are able to meet the needs of their clients and ultimately ensure the safety of the public (Sharpless & Barber, 2009). Awareness of one’s own competence facilitates educated decisions about referrals to accept, clients to refer on, and when to engage additional supports or expertise to best meet the needs of the clients (Loades & Myles, 2016). Assessment of competencies has also been indicated more broadly in career planning (Cooper & Rothmann, 2013).

Whilst the importance of maintaining competence is noted in both the literature (e.g., Barnett et al., 2007; Loades & Myles, 2016; Sharpless & Barber, 2009) and the relevant ethical guidelines (e.g. Australian Psychological Society, 2007), how psychologists are to meet this obligation of monitoring their competencies is unexplored. Despite the requirement to engage in professional development and supervision, the question remains as to whether these activities are ensuring both maintenance and development of the required psychology competencies. As a result, self-assessment is a skill that is useful for both psychologists and the broader health disciplines, with self-assessment being “one of the basic skills implicit in our current models of self-directed learning, continuing education and self-regulation” (Eva & Regehr, 2007). However, this in itself poses significant issues, with psychologists at times failing to adequately evaluate their competencies (Knapp et al., 2017a). Indeed, it has been noted that whilst “effective psychotherapists know their strengths and weaknesses … such self-appraisals may be one of the hardest tasks that psychotherapists have.” (Knapp et al., 2017b, p. 165).

There is both significant overlap, and yet clear distinction, between the complex cognitive, emotional and behavioural processes of self-assessment and self-reflection. The American Psychological Association (APA) defines self-reflection as the “examination, contemplation, and analysis of one’s thoughts, feelings, and actions” (2020). A definition of self-reflection within the context of clinical practice is offered by Knapp et al. (2017a): “self-reflection is a deliberate metacognitive process involving self-observation of thoughts, feelings, attitudes, and behaviours, with as much objectivity as possible” (p. 167). Knapp et al. (2017a) also highlight the way that this process of self-reflection is then utilised by psychologists: “ … adaptive self-reflection looks at specific behaviours or experiences and leads to insight and changes in one’s behaviour or attitudes” (p. 167). Self-reflection is considered to be one of many core competencies for psychologists, alongside other competencies such as assessment and intervention (APAC, 2019; British Psychological Society, 2019). In contrast Eva and Regehr (2007) offer a definition of self-assessment within the medical field as:

… a process of personal reflection based on an unguided review of practice and experience for the purposes of making judgments regarding one’s own current level of knowledge, skills, and understanding as a prequel to self-directed learning activities that will improve overall performance and thereby maintain competence. (p. S81)

Further to this, Eva and Regehr (2007) note that self-assessment may extend beyond “an ability to rate one’s strengths and weaknesses” (p. S82) to also include “situation-specific awareness” (p. S82) whereby practitioners are able to identify and resolve gaps in skills and knowledge pertinent to the issue at hand. These definitions align with Boud’s (1995) earlier conceptualisation of self-assessment as having two stages: “establishing criteria and judging work in the light of them” (Boud, 1995, p. 12). Whilst there is significant overlap in self-reflection and self-assessment as cognitive processes exploring the experience of work, self-assessment has a central focus on one’s competencies.

Boud (1995) recognised the importance of self-assessment in developing “effective and responsible learners who can continue their education without the intervention of teachers or courses” (p. 13). The paramount role of self-assessment for psychologists is evident when we consider the way in which psychologists’ competencies continue to develop beyond completion of tertiary training and throughout the career (e.g. Rodolfa et al., 2005), and the requirement for psychologists to often apply such competencies without direct observation from supervisors. Like many health professionals, psychologists often practice without direct supervision and thus require a degree of self-monitoring to ensure continued quality practice. However, little is known about what self-assessment looks like for psychologists. The concept of metacognition offers a useful lens through which to understand psychologists’ experiences of self-assessment. Definitions of metacognitions have ranged from “thinking about the contents and processes of one’s cognition” (Winne & Azevedo, 2022, p. 93) to a more active definition offered by Martinez (2006) of “the monitoring and control of thought” (p. 696). In applying a metacognitive lens to understanding self-assessment for psychologists, we may begin to understand how responses and appraisals of the experience of self-assessment serve to shape self-assessment.

Further, whilst it has been asserted in other health disciplines that self-assessment may be enhanced through the use of structured measures of competence (Sheridan, 2021), there is scant research that explores the utility of structured measures of self-assessment for psychologists. This may be related to the dearth of structured measures for psychologists across their career trajectory. In response to this gap, Rice and colleagues (2025) developed the Competencies of Professional Psychology Rating Scales (COPPR Scales) which provides a multi-dimensional measure of psychological competencies based on the Australian Psychology Accreditation Council (2019) professional competencies for general psychologists. Given the emphasis on competency assessment and development for psychologists and health professionals more broadly, it is likely that more measures mapped to the specific psychology competencies as stipulated by professional bodies will emerge with time. Structured measures, such as the COPPR Scales, are needed to facilitate a comprehensive assessment of competence within self-reflection. This provides the ability for psychologists to undertake a systematic and holistic evaluation of their competencies across all domains of practice. This also facilitates an assessment of change over time, such as with professional development activities and throughout the career trajectory. However, to date, there is little research exploring the cognitive process of self-assessment for psychologists, or how this may be aided with a structured self-assessment tool. As a result, the current research sought to answer the following research questions:

1. How do psychologists currently self-assess their competencies?
2. What utility or value does a structured measure offer psychologists for self-assessment?
3. What are barriers or limitations for psychologists when using a structured measure for self-assessment?

In exploring these research questions, this study expands on the current psychology literature to explore what factors facilitate and hinder the self-reflection and self-assessment processes. This is an important step towards further research as to how best address these barriers in order to facilitate effective self-reflection and self-assessment across the psychology career trajectory.

## Method

### Design

A qualitative research design allowed for the in-depth exploration of the process of self-assessment and the barriers that arise for psychologists. Specifically, reflexive thematic analysis (Braun & Clarke, 2022) was chosen due to its emphasis on reflexivity and fluidity in qualitative analysis. Reflexive thematic analysis was used as a means “to achieve an understanding of patterns of meaning from data on lived experiences” (Sundler et al., 2019, p. 736). Indeed, a methodology based on reflection was fitting for research aiming to understand the nature of such cognitive and metacognitive processes as self-assessment in the context of the psychology discipline, as it allowed the researchers to utilise their own understanding of self-assessment whilst being open and curious to participants’ understanding and experiences.

#### Participants

All participants held current registration as a psychologist in Australia. The study was advertised at various professional forums and websites. A total of 12 participants engaged in the study, and of these, six held general registration, and six held general registration with additional endorsement in the area of clinical psychology. The sample was predominantly female, with nine participants being female and three participants being male (See Table 1 for summary of participant characteristics.) With regards to sample size, there has been significant conjecture in the literature for what is an adequate sample in qualitative research. Whilst some conventions have been suggested (e.g., 12 participants as suggested by Guest et al., 2006), more recent focus has been on information power whereby the depth of information gained from interviews is considered more conducive to rich analysis, rather than the number of interviews conducted (Malterud et al., 2016, 2021). Information power was maximised through using interviews rather than focus groups, and through a semi-structured approach which allowed for flexibility in question order and probing to foster responses with depth. Information power was also maximised through the focused study aim and the specific sample characteristics, in that as the participants were psychologists with training in self-reflection, they were expected to be somewhat familiar with the topic at hand and able to meaningfully contribute with relative ease.

**Table 1:** Sociodemographic characteristics of interview participants

Participant	Sex	Years of registration	Registration status
1	Female	20*–*30	General
2	Male	20*–*30	General
3	Female	0*–*5	General
4	Male	6*–*10	Clinical endorsement
5	Female	10*–*20	Clinical endorsement
6	Female	6*–*10	General
7	Female	6*–*10	Clinical endorsement
8	Male	0*–*5	Clinical endorsement
9	Female	6*–*10	Clinical endorsement
10	Female	30*–*40	Clinical endorsement
11	Female	10*–*20	General
12	Female	6*–*10	General

Note. Registration status aligns with that of the Psychology Board of Australia (2024a), whereby “general registration allows psychologists to work in any area of psychology that is within their scope of practice and to use the tile ‘Psychologist’”, having completed a minimum of six years of education and supervised practice, and passed the National Psychology Exam. Psychologists in Australia can obtain an area of practice endorsement in various areas (e.g., clinical psychology, forensic psychology etc.) in addition to general registration, which serves to “identify practitioners within a profession who have undertaken additional qualifications” (Psychology Board of Australia, 2024b).

### Procedure

Of the 12 interviews conducted, nine were conducted via Zoom teleconferencing software and three were conducted in person where geographically possible and preferred by participants. Participants provided informed consent online through Qualtrics at commencement of the interview with the opportunity to ask the researcher any questions. Interviews were recorded and then transcribed verbatim by the first author.

#### Interview

Interviews allowed psychologists to share their experiences of both unstructured self-assessment, and the experience of completing a structured measure of competencies and the perceived utility of this. Interviews have been used within the field to understand the intricacies of self-reflection and reflective practice in psychologists (Carmichael et al., 2020; Fisher et al., 2015). and the present study aimed to use the richness of information shared in interviews through participants’ self-reflections in order to develop a richer understanding of self-assessment in psychologists.

Following consent, the first stage of the interview asked participants to provide some general information about their professional roles before moving into questions about unstructured self-assessment (e.g., “Tell me how you currently see your areas of strength in terms of your competencies”; “Tell me about how you think about your competency and how you assess it.”). Participants then completed the COPPR Scales (Rice et al., 2022) as a structured self-assessment of competencies, before answering questions about the measure (e.g., “Firstly, could you tell me generally how the experience of completing the COPPRs was for you?”) and reviewing their results which were automatically tabulated via Qualtrics and presented upon completion of the measure (e.g., “Looking at your results on the COPPRs, how accurately do you feel the domain subscales reflect your competency areas?”). There were further questions about the COPPRs as a tool for self-reflection (e.g., “What do you think about including a structured measure of competencies such as the COPPRs into your self-reflection activities?”), and some short questions about career development and competencies (e.g., “Assessment of competencies is often the first step in career planning. Do you consider your competencies in your current plans?”).

#### Competencies of Professional Psychology Rating Scales (COPPR Scales; Rice et al., 2025)

The COPPR Scales (Rice et al., 2025) are a newly developed standardised measure of psychologist competencies, as mapped to the Australian Psychology Accreditation Council (2019) standards for registered psychologists. The self-report version of the measure (COPPR-S) is comprised of 81 items across 11 domains, with each domain acting as a subscale. These domains include: scientist-practitioner, cultural responsiveness, working across the lifespan, communication and liaison, clinical interviewing, counselling micro-skills, formulation and diagnosis, assessment, intervention, ethics and self-reflective practice. In a recent pilot study, the COPPR Scales have demonstrated content validity and both strong convergent and divergent validity, as well as sensitivity to differentiate between early career and more experienced psychologists (Rice et al., 2022). Banner et al. (2023) recently found the self-reflection subscale of the COPPR Scales to be significantly positively correlated with the self-reflection subscale of the Self-Reflection and Insight Scale (Grant et al., 2002), as well as the 12-item version (Silvia, 2021) and 14-item version as tested with Australian psychologists (Banner et al., 2023). Items are rated using a 7-point Likert scale, ranging from 1 (Not Yet Competent), to 7 (Expert). Detailed descriptors of each rating point are provided to participants, and this is provided for each subscale of the measure in order to increase chances of accurate scoring. Scores can be interpreted as a mean item response score for both the total score and each subscale. Participants completed the COPPR Scales using Qualtrics during the interview, and were instantly provided with a copy of their results in a table with mean scores for each subscale to which they could refer for the second part of the interview.

### Data analysis

Following transcription by the first author, each transcript was then coded by the first author with the assistance of NVivo 12 Plus qualitative analysis software. NVivo 12 Plus served two methodological purposes: a means of data organisation to increase ease of sorting and refining codes across the twelve transcripts rather than analysis in and of itself, and also as a crucial component of the audit trail of analysis (Franklin et al., 2019). Reflexive thematic analysis (Braun & Clarke, 2022) was used to analyse data, using an inductive approach that allowed the researchers to best understand the experiences of the participants beyond the limitations of the existing literature. Initial codes were reviewed and refined multiple times to ensure they best captured the data and that each code added unique value to the analysis. Initially all codes were reviewed and those which were either simply quotations or better captured by an existing code were identified. Transcripts were also reviewed multiple times with codes being added and deleted during this process as the first author further reflected on the meanings shared by participants. Alongside this process, codes were clustered based on shared meaning, which also led to the both the refinement of existing codes and development of some additional codes as the researchers identified a more latent meaning as familiarity with the data increased. Codes continued to take shape as code clusters were developed into themes based on shared meaning and patterns across the data. Initially, the researchers developed themes around metacognitions relating to process of self-assessment and then also about the self; however, as codes were refined the thematic maps were organised into two parts: one part focussing on themes relating to the process and utility of self-assessment, and another on themes encapsulating the barriers to self-assessment. Thematic maps were drafted across a number of versions with shifts in both the thematic structure (e.g., themes and sub-themes), and the naming of themes themselves to best capture the meaning expressed by participants.

### Reflexivity

At all stages of data collection and analysis, immersion and reflexivity were at the forefront for the researchers. Researchers were guided by a relativist ontological and epistemological approach, using reflexivity to recognise the role and input of the researchers throughout the course of the study. All authors were registered psychologists and/or psychology academics, were familiar with both unstructured and structured self-assessment (including the COPPR Scales), and had varied experience in qualitative research and reflexive thematic analysis. Prolonged engagement can be useful for allowing reflexivity (Franklin et al., 2019) and establishing trustworthiness (Nowell et al., 2017), which occurred as data collection continued over the course of 20 months. Other means of increasing trustworthiness included the first author keeping a reflective journal, and key points were shared with other authors through peer debriefing as the study progressed. Peer debriefing was also used across the study design, data collection and analysis to assist with both adherence to the thematic analysis methodology and also to facilitate further reflection, such as in reviewing the coding process and thematic diagrams. Peer debriefing includes a peer acting as a “critical detective” (Janesick, 2015) in reviewing the study across various stages and providing feedback to improve the overall quality of the study.

## Analysis

**Fig. 1:**
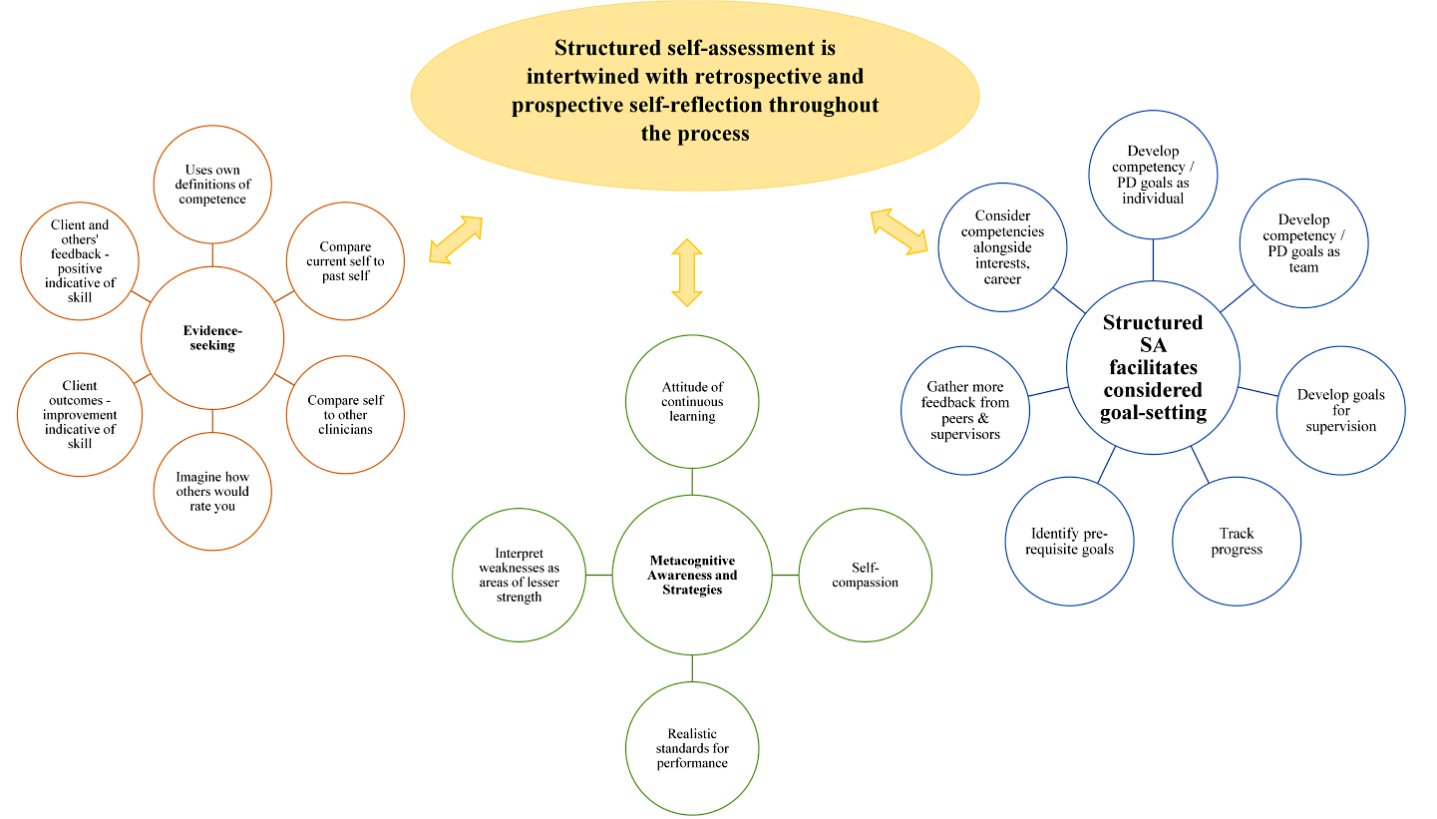
Thematic map: metacognitive processes and helpful cognitions that aid self-assessment and the use of a structured self-assessment measure

**Fig. 2:**
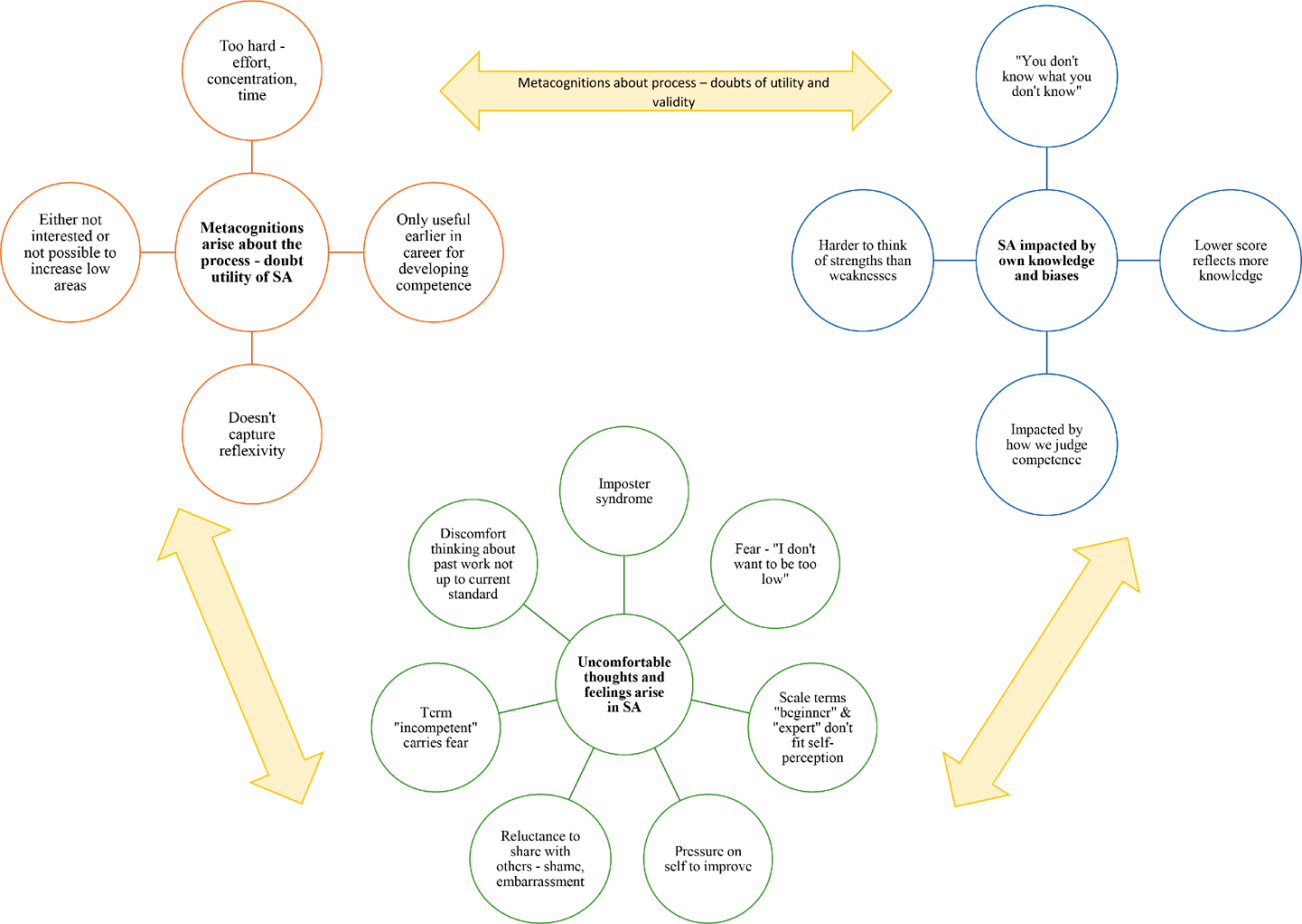
Thematic map: barriers to self-assessment

Two thematic maps were developed in response to the research questions, with one addressing the process of self-assessment (Fig. 1) and the other barriers to self-assessment (Fig. 2). The first thematic map was developed in response to the first two research questions: How do psychologists currently self-assess their competencies, and what utility and value does a structured measure have for self-assessment? This thematic map was loosely defined as a “process of self-assessment” as themes shared a commonality of describing the process of self-assessment for psychologists, including the utility and value of structured self-assessment. This map included one theme and three sub-themes. The second thematic map was developed in response to the last research question: What are barriers or limitations to using a structured measure for self-assessment? This thematic map was loosely defined as a “barriers to self-assessment” thematic map given the commonalities that each of the three themes explored. Together these two maps illustrate the complex interplay between self-reflection and self-assessment, and the metacognitive natures of self-assessment.

### Thematic map 1: process of self-assessment

Participants described a complex internal process of self-assessment, which was captured through one theme that structured self-assessment is intertwined with retrospective and prospective self-reflection throughout the process, and three sub-themes including 1) evidence-seeking, 2) metacognitive awareness and strategies and 3) structured self-assessment-facilitates considered goal-setting. Together, these illustrate the way in which a number of cognitive processes (e.g., evidence-seeking based on definitions of competence) interact with metacognitions (e.g., interpretations about the results and their implications), and lead to the deliberate development of goals when psychologists are engaging in structured self-assessment.

#### Structured self-assessment is intertwined with retrospective and prospective self-reflection throughout the process

Participants described using self-reflection across all points of self-assessment, including reflection of past work experiences, reflection of thoughts and feelings in the moment whilst completing the measure and interview, and then shifting attention to future steps when reviewing the results and considering goals. For example, P1 articulated some of their thoughts as they were completing the measure (e.g., *“I work so differently now…”*) and noted how their results on the measure “*affirms*” their current professional development goals and that “*I really do want to make it happen”*. This offers an insight into the way in which introspective information interacts with the results on self-assessment scales when developing goals. Without self-reflection and the information that this brings about our own motivations, interests and values, goals from self-assessment would simply reflect the results, however there is a much more complex interplay occurring.

##### Evidence-seeking

Participants described a process of evidence-seeking in order to guide and inform self-assessment. Evidence was sought in a range of ways. A commonly reported form of evidence-seeking for participants was having their own benchmarks for self-assessment, which they would apply to a past instance of executing a specific competency. P4 articulated this process: *“ … just thinking back to, you know, trying to find examples and think through kind of recent examples…, just trying to cast your mind back to bits and pieces of work.”* These reflections were then appraised according to certain standards. P1 described applying a set of internalised criteria for assessing their competence in a given situation: *“ … I’m doing those sort of checklists in my head because of how if I’ve … got experience in that particular thing, so I’ve done it lots and lots of times.”* Despite the heterogony of responses from participants about how they define and evaluate their competence, there was a shared sense of engaging in reflection to draw evidence for competency, with P11 articulating this:

Assessment is something that I considered I did really well like, I … remember going into Masters and it was like I labelled it as one of my strengths. And then when I found out more about assessment and self-reflected on how I’m doing assessments and how I’m writing reports and doing all that. I was like, wow, no, I’ve got so much more that I need to learn and to improve on.

This suggests a sort of internalisation of competency standards and a process of comparing performance to these internal benchmarks.

Participants also described part of the evidence-seeking as considering feedback from clients and others’\ (e.g., peers from both psychology and other disciplines, their supervisor and supervisees even), as well as client outcomes. P6 described that in addition to supervisor feedback about their strengths, client observations are an important piece of evidence that informs perception of strengths and weaknesses: *“You know seeing improvements in my clients, them coming and saying yes that was really helpful … seeing them change in ways that they’re thinking about things and responding to things.”* P1 also described evidence of competencies as when clients *“keep coming*”. P2 extrapolated on this and noted how they relied on client assessment to gauge their competency, in part due to the absence of a standardised measure to do so: *“And there are no external independent measures at the moment that you can sort of say, well, these are my strength areas or my less strong areas or struggles. I’m not sure about that. I … think. I guess I … picked this up from my clients….”* Client feedback and perceived indicators of client satisfaction or progress (e.g., clients returning) appear to be strong indicators of competency for psychologists when engaging in self-assessment.

Reflections about other clinicians also appear to shape perceptions about competence and form part of the evidence-seeking process. For example, P5 described how their perception of competency was shaped not only by client feedback, but by the specific clients with whom they were willing to work compared to other clinicians:

… I think when it comes to complex cases. It’s something that you just kind of observe when nobody else is willing to take them places that might not necessarily concern me, and it’s like, okay, well, this must be a strength. Nobody else is willing … to see these people or they’re feeling anxious about it then, you know. And I’m … not, I guess put off by that then that feels like a strength, I suppose.

This sense of comparison to other clinicians was echoed by P9, and suggests that psychologists use their perceptions of other clinicians to develop their own self-assessment.

##### Metacognitive awareness and strategies

Participants described many cognitive strategies to facilitate ongoing engagement in self-assessment, suggesting an overall metacognitive awareness of their experience and efficacy of self-assessment. In particular, the variety of strategies described by participants suggested a personalised component to self-assessment, where psychologists may be monitoring their experience of self-assessment and drawing on strategies to continue engagement in self-assessment despite barriers to doing so. For example, participants described drawing on more overarching attitudes, such as that of continuous learning and being goal-oriented. P2 articulated the attitude of continuous learning, including an acceptance that learning can come when things do not go well:

Yes, I. see areas there where I feel that I am proficient. However, there are areas I … do need to learn. And my attitude … has always been lifelong learning and lifelong learning for me has certainly been yes in that formal environment absolutely, but also just learning new things every day. … And if something doesn’t go well or something does go well, then I’ve learned something.

This attitude was similar to that expressed by P8, who clearly expressed the value in continuing competency development: *“ … competency building activity is actually really critical to strong kind of sense of self and I think I really enjoy helping people and I think that’s … good, and I think the realisation of what feeling competent does for your mental health…”* Interestingly, this appears to allow for the simultaneous appreciation of both strengths and weaknesses, with P8 also describing *“ … I’m proud of my things that I’m better at and I can see pretty clearly on where could I do some work…”,* with this approach allowing for a balanced perspective of both strengths and weaknesses and the *“more of a concrete direction*” (P8) in terms of next steps. Through facilitating the consideration of both strengths and weaknesses, positive attitudes towards and perceived value of continued competency development appears to allow for richer self-assessment.

Participants described the way in which an awareness and at times challenge to self-cognitions aids self-assessment, in particular through adopting a more compassionate approach to the self. P12 described the role of self-compassion in challenging initial responses to results:

Yeah, I guess some of that is initial feelings of I need to keep improving and a little bit of pressure on myself to keep improving … but then if I sort of take that sort of negative … like I was talking about before of, you know, being a bit kinder to myself.

This kindness is likely related to having realistic standards for performance, which appeared to be helpful for participants. Compassion for oneself as a constantly developing practitioner appears to foster psychological flexibility to accurately self-assess without adhering to unrealistic standards for practice (e.g., to be an “expert” early in career, or to be greater than “competent” across all domains). For example, P7 reflected on their results: *“I think to me it looks pretty good I think, like based on where I am. I think I’m seven years out of from my Masters, so I feel like that’s probably where I should be at and I feel like if you’re at a 7* [expert rating on the COPPR] *in everything then you’re either narcissistic or you don’t feel you’ve got any areas to develop in…”* This suggests realistic standards for performance both in terms of considering one’s career stage and experience, and also having an acceptance that it is not possible to be an “expert” at all competencies. This is similar to an expectation of being competent rather than “incompetent” across all competencies, which P3 reflected *“made me feel good.”* It appears that self-assessment can bring a number of positive emotions and thoughts. Interestingly, P4 described how the nuanced nature of the structured assessment allowed for helpful metacognitions:

… Obviously it does really get you thinking, specifically, you know, like it how it breaks it down. I think … a lot of time you might think broadly around … for instance … how are my intervention skills, but really breaking it down into so many categories, you know you can see areas of strength and weakness within. So you didn’t have to kind of think, am I good at this or bad at this, it was kind of like well it’s a mixed bag, is something you can kind of take away from it, I suppose.

This contrasts the potential of black-and-white cognitions of being all good or all bad at a competency, and the way in which structured assessment of the nuances of a competency facilitates challenging these cognitions to allow for more nuanced and meaningful self-assessment.

##### Structured self-assessment facilitates considered goal-setting

Goal development appears to be more effectively facilitated through structured self-assessment as opposed to unstructured self-assessment, through the more thorough and deliberate cognitive process that structured self-assessment allows. For example, after completing the COPPR Scales, many participants discussed how this assessment would prompt them to potentially develop competency goals as individuals (e.g., for supervision, for professional development activities) and also as a team in their workplace (e.g., a shared goal to increase skills in assessment). A number of participants described how completing the COPPR Scales would result in the development of SMART goals (P7), with some participants articulating newly formulated goals upon reflection of the results. For example, P3 noted that whilst they would not shift their CPD plans specifically, they would like to engage in more time self-reflecting each week. Interestingly, increasing the structure of self-reflection was shared as a goal by a number of participants, including through increased time engaging in reflective journaling (P7). There was also a sense that the efficacy of goal development could be further enhanced through reviewing the results in supervision, as P4 articulated:

Just fleshing out those conversations and getting that specificity around it … so that you’re getting kind of really good specific goals that are measurable as opposed to just kind of a broader goal of … this year I want to focus on assessment.

Supervision appears to also have a purpose of providing additional context to the results, as participants spoke to the need for seeking more feedback, and how a richer picture would develop from this, with P10 likening it to other forms of assessment used in a clinical setting:

And I think self-report has to be … it’s the same with your onus when you’re doing assessment, you don’t rely on your self-report. You try and get other information and put that together with the self-report scale that kind of would confirm … or disconfirm or kind of get you wondering about something and wanting more information.

This conveys a sense that structured self-assessment, when including other perspectives, may assist with weakening the impact of confirmation bias on perception of competencies.

Self-assessment was also discussed as a valuable tool to identify pre-requisite goals, as in steps that needed to be addressed prior to others. For example, P4 discussed how they had already developed goals around assessment and diagnosis, however *“I can see how if I had done this first it’s identified something else that would be quite important … so I think it … would have strengthened the work I’ve already done in developing goals for the year.”* Participants also shared an interest in tracking progress, for example as part of supervision (P10) or after engaging in professional development opportunities (P2). Tracking progress may be aided through a structured measure which allows for quantifiable observation, rather than relying solely on unstructured self-assessment.

Structured assessment also appears to foster both a broader assessment of all competencies, and a more nuanced assessment of each individual competency, compared to unstructured self-assessment. P12 articulated this discrepancy:

You know, I tend to sort of have that little bit of an, just an internal think about things … depending on my current caseload and what’s happening for me at the moment. I think doing this, I was able to get a bit more both specific in terms of my skills, but general in terms of … I’m contradicting myself by … saying both specific and general, but … both.

In a similar vein, P7 noted it was out of the norm to assess the whole range of competencies:

I think when you’ve been practicing for so long you don’t really reflect on your competencies as much as a student or as a training psychologist straight out of uni so it’s good to come back to that as that’s at the core of your whole practice.

Interestingly, P7 notes the importance of competencies, yet the tendency for clinicians to not assess these specifically as clinicians continue along their career trajectory, indicating that perceived importance may not be enough to ensure regular and effective self-assessment. Overall, participants shared a number of ways that structured self-assessment leads to the effective development of goals across all stages of the career.

### Thematic map 2: barriers to self-assessment

Participants described two key themes relating to metacognitions about the process of self-assessment, including 1) doubts of utility and 2) self-assessment is impacted by own knowledge and biases. Participants also described 3) uncomfortable thoughts and feelings arise in self-assessment. Together, these three interrelated themes interact as barriers to participants engaging in self-assessment, with participants outlining doubt as to whether the results of self-assessment are accurate or actionable, and find the process challenging in terms of cognitions and emotions.

#### Doubts of utility

Some participants expressed doubt about the utility of a structured measure of self-assessment, including some pragmatic concerns about the time and concentration required, but also some deeper concerns that structured self-assessment does not capture reflexivity and is only useful earlier in career for developing competence. For example, P2 shared the way in which reflective journaling facilitated reflexivity more so than a structured measure of self-assessment:

… journaling to me is about this is what I am feeling. This is what is going on within me. This is how this person, when they said that, that triggered me. Sure, this is how I responded to that trigger….

It appears that the perceived value of structured self-assessment measures is limited as it does not align with this participant’s perception of self-reflection. Other participants also noted that structured measures are more useful earlier on in their career, as articulated by P1: *“Because I think once you get to a certain level in your career, it would just become another tick box thing unless it was meaningful. And for [it to be] meaningful, it would need to be more nuanced for me.”* This indicates a sense that self-assessment has greater value earlier in career, as later on in career skill development becomes more nuanced.

Some participants also highlighted that they are either not interested, or feel it is not possible within their current work settings, to remedy areas of weakness. This manifested for some as an interest in further developing strengths. This sense was shared by P5:

… I think in in terms of my career, I’m probably more likely to move towards my strengths and to feel like I want to amplify the things that are actually probably down that advanced or expert section. So you know playing into that more as opposed to identifying areas of need that I want to sideline…

Other participants indicated that they were restricted by their work circumstances, such as the scope of their roles or funding agreements reduce their ability to engage in changes to service delivery to build competencies (e.g. thorough client assessment or long-term intervention). This suggests that the perceived value of self-assessment is impacted by one’s goals and perceived scope to develop their competencies.

#### Self-assessment is impacted by own knowledge and biases

Participants also raised a broader theme that self-assessment is biased by one’s knowledge of the competency. Overall, there was a sense shared by some participants that their self-perception and their competence were two separate things, with the latter being impacted by the former so much so that it severely limited the validity and thus utility of self-assessment. For example, multiple participants noted “*you only know what you know*” and “*you don’t know what you don’t know*” indicating that self-assessment is limited by knowledge, with P11 extrapolating on this further when describing her growth in knowledge and skills in assessment:

So I probably would have rated myself as a 5 back then. But now I know more again, if I had rerated the past self, I would not have been 5 … I’ll still be five now. But now I consider my past self not.

P10 also noted the interaction of “*the self*” as a whole, rather than limited to their role as a clinician:

… I think this is about the self, you know, and how you rate yourself and then this is the thing I never know like how I see myself is not, how do I know how other people see and experience me, like it’s very one sided.

Together these suggest that clinicians have a sense that self-assessment is hindered not only by their professional knowledge, but also their own personal biases and blind spots.

Some participants also noted the role of a bias to think of weaknesses rather than strengths. Interestingly, a number of participants spoke of this in the context of cultural responsiveness in particular, with P5 noting:

… I think I just am aware that there’s just so much that I don’t know that I think even though I’ve, … got a willingness and a strength to want to know how to respond appropriately to somebody’s culture, I just know there’s never, you’ll never be an expert I think in somebody else’s experience or somebody else’s culture, unless it’s your own culture … and … even then, you’ve got a bias there.

Similarly, where participants described how they judge their competence, some also noted that how competence is judged impacts self-assessment. For example, P9 noted how comparing herself to other clinicians was probably unhelpful*: “ … I think that my kind of assessment of what my skills are is a comparison against clinicians who that possibly isn’t a fair comparison because they have skills I don’t have and I have skills that they don’t have.”* Overall, psychologists voiced an interest in the way in which self-assessment is impacted by factors related to themselves, such as their existing knowledge base and personal biases.

#### Uncomfortable cognitions and emotions

Another barrier to self-assessment related to unhelpful thoughts and feelings that arise in self-assessment. That is, participants described how as they were reading items while completing the COPPR, they engaged in cognitive processes to determine where they should indicate their competence falls on the Likert scale. They also reported that in completing the measure, they at times experienced some uncomfortable thoughts and feelings, in particular about themselves and their competence. For example, participants described how the anchors of the scale (which includes terms such as not yet competent, beginner, expert) and also the word “incompetent” itself were “*value-laden labels*”. Whilst this feedback was specific to the COPPR Scales, it could be anticipated that other structured measures around competence may also incite such responses. Other participants described specific cognitions in more detail including “*I don’t want to be lower*” (P12) and a general sense of imposter syndrome. When talking about how imposter syndrome arises through their engagement in self-reflection, P3 described *“ … a little bit of a fear of am I reflecting enough and it goes back into that whole clinician imposter syndrome type thing, like am I doing it right?”.* Participants also raised a number of uncomfortable feelings including “*reluctance*” around rating themselves as an expert as this term did not fit for them, some discomfort about the possible inaccuracy of the results *(P8: “ … I think my results are only as accurate as my knowledge of those domains and that’s what, I suppose, that’s what sits a bit uncomfortably for me, as I’m answering it I tried to do the best I could…”)* and a lot of uncomfortable thoughts and feelings around performance and need to improve including as P12 described *“initial feelings of I need to keep improving and a little bit of pressure on myself to keep improving…”* Also evident was that reflection led to some discomfort when considering past skills, as articulated by P11: *“ … it’s so funny. Back then I look at my work back then … I thought that was good. It wasn’t bad, but it was just you know.”* It appears that self-assessment can be a challenging process for clinicians in terms of both cognitions and emotions and what it means for them in terms of their past practice, their current practice, and what needs to occur to continue to improve future practice.

## Discussion

The present research sheds light on the metacognitive process of self-assessment, including structured self-assessment, and highlights the barriers that psychologists face in doing so. The first thematic map highlighted the role of self-reflection throughout self-assessment, and identified that the process of self-assessment is guided by “rules” for how one self-assesses, and also definitions of competence. Analysis also suggests that this process is aided by a metacognitive awareness of the experience of self-assessment, with psychologists utilising helpful cognitive strategies about both the self and self-assessment in general. Thirdly, it appears that structured self-assessment is a deliberate, considered process which participants felt would lead to the effective development of goals more so than unstructured self-assessment. A second thematic map identified barriers to engaging in self-assessment, including doubts about the utility of self-assessment and a belief that self-assessment is impacted by own knowledge and biases (and, therefore, is unreliable). Further, when engaging in self-assessment, uncomfortable thoughts and feelings arise which act as a barrier to engaging in self-assessment.

### Implications

All participants shared a value for competence development and self-assessment as part of this process. However, how psychologists experience self-assessment had particular nuance, in terms of both recognising the value and necessity, but also its limitations. It appears psychologists have significant metacognitive awareness of self-assessment, including being very conscious of biases and the possible shortcomings of one’s self-perception. The role of broader motivations and attitudes towards goals and learning as highlighted by the current participants may extend beyond the scope of their work, and also be a personal quality. This overlap between the self and the self-as-therapist has been hypothesised to be a component of therapist skill development (Bennett-Levy, 2006).

Interestingly, there was overlap in participants’ descriptions of self-assessment existing definitions of self-reflection including Schön’s (1983) reflection-on-action, and Killion and Todnem’s (1991) reflection-for action. For example, participants clearly articulated reflecting on past practice (as they were not currently practicing at the very moment of being engaged in the research), and also discussed how they would use their reflections and results to formulate future actions. This adds to the current dialogue around the overlap and distinctions between self-assessment and self-reflection (e.g. Desjarlais & Smith, 2011; Eva & Regehr, 2005). Further, the current research suggests that structured self-assessment measures may assist in meaningful reflections-for-actions in terms of the development of clear goals.

The identification of helpful and unhelpful cognitions arising in self-assessment is a significant contribution to the field. Whilst some measures of self-reflection in the health sciences have been developed (see Ooi et al. (2020) for a review), there are no existing measures capturing specific cognitions related to self-reflection and self-assessment, likely due to the limited research using a metacognitive lens to identify the cognitions people experience as they engage in both self-assessment and self-reflection. A self-report questionnaire including common helpful and unhelpful cognitions about self-assessment would allow practitioners to further increase their metacognitive awareness of self-assessment, which may lead them to using helpful cognitive strategies for maintaining effective engagement in self-reflection despite the barriers to doing so.

Further, whilst there are key differences between self-reflection and self-assessment (e.g. Desjarlais & Smith, 2011), it is possible that the current research may add to current conceptualisations of self-reflection within the health sciences. For example, contemporary models of self-reflection typically emphasise a step of identifying what the clinician needs to do differently (e.g., REFLECT model; Lawrence-Wilkes (2014)), and it is likely that this cognitive process would share the barriers identified in the current analysis, such as imposter syndrome and pressure on self to improve, whilst it may be aided by more helpful cognitions shared by participants in this research, such as an attitude of continuous learning and goal development.

An interesting component of the research was the detail that participants shared about their “rules” for self-assessment and how their own definitions of competency impact this. Indeed, Boud (1995) noted that an essential component of self-assessment is recognising the benchmarks for performance. In this case, psychologist self-assessment relies on a definition of competency, which clearly varies across individuals. Psychologists may benefit from direct guidance about the standard for competency so as to accurately self-assess. However, the findings from this study suggest a benefit from self-assessment of competencies across all stages of the career trajectory.

The research supported the utility of structured self-assessment measures such as the COPPR Scales (Rice et al., 2022), both in terms of more simultaneously specific and broad competency assessment, and in developing goals. Further research into how structured self-assessment measures can be used in a supervisory context may assist in expanding understanding how these goals are conceptualised and developed as a result of completing structured self-assessment measures, and whether competencies are developed more effectively as a result of engaging in structured self-assessment measures as opposed to unstructured self-assessment.

### Limitations and directions for future research

Whilst a qualitative approach allowed for a rich understanding of self-assessment for psychologists, further research may explore the transferability of the findings to both other psychologists and related health disciplines. For example, future research may adopt a developmental lens to explore whether provisional psychologists whom are earlier in their training may share this experience of the processes and barriers of self-assessment, or whether there are particular nuances to self-reflection and self-assessment in the early career stages. It would be of relevance to other developmental models of supervision (e.g. Stoltenberg & McNeill, 2010) to explore whether metacognitions towards self-assessment may shift across the developmental trajectory, as this may assist with more targeted and specific interventions (e.g., supervisory approaches) to assist practitioners in overcoming barriers to engaging in meaningful self-reflection. Beyond psychology, it would also be of note to explore whether other health disciplines experience similar processes and barriers to self-assessment, given the recognition that “effective self-assessment is a critical skill for any health professional” (Eva & Regehr, 2007, p. S81).

In a similar vein, the present study’s methodology consisted of a retrospective approach to understanding self-assessment in terms of encouraging psychologists to reflect on their practice outside of the interview. Upon researcher reflection of the transcriptions, it became apparent that it is difficult to discern at times in the interviews whether participants were sharing their cognitions around self-assessment as they arose in the course of the interview, or reflections on self-assessment that they had previously experienced. It would be of significant interest to explore whether “reflection-in-action”, as described by Schön (1983), where practitioners engage in reflective processes in vivo, are also impacted by the same or similar metacognitions as identified in the current research. Thus, research that investigates the process of reflection in vivo may offer further insights.

Whilst cognitive-behavioural models have been applied to learning acquisition for therapists (Bennett-Levy, 2006), due to the limited research on metacognitive barriers to self-assessment to date, there has been little application of otherwise evidence-based approaches to cognitive change specific to clinicians in terms of self-assessment. Lilienfeld and Basterfield (2020) have suggested that reflective practice can greatly benefit from the wider psychology literature, and it follows that self-assessment may as well. Future research in this area would be of great interest to the field and may aid in the development of tools to support psychologists in developing a healthy, helpful approach to self-assessment and self-reflection which ultimately facilitates further competency development.

### Conclusion

Overall, this research offers the first known qualitative account of both metacognitions related to self-assessment, and also psychologists’ experience using a structured self-assessment measure. This has significant implications for psychologists in self-identifying their barriers to self-assessment, and also may aid supervisors in guiding supervisees towards more effective self-assessment. This research paves the way for the future development of approaches to continue to support psychologists in meeting ethical obligations around self-assessment and maintaining competence.

## Data Availability

Data may be available on reasonable request from the corresponding author subject to ethics approvals.
